# Revealing the photocatalytic dissociation of water molecules on rutile TiO_2_ surface *via* hybrid functional based linear response time-dependent density functional theory

**DOI:** 10.1039/d5sc02736e

**Published:** 2025-08-22

**Authors:** Lei Wang, Xiaofeng Liu, Qunxiang Li, Jinlong Yang, Wei Hu

**Affiliations:** a Department of Chemical Physics, and State Key Laboratory of Precision and Intelligent Chemistry, University of Science and Technology of China Hefei Anhui 230026 China liqun@ustc.edu.cn; b School of Physics, Hefei University of Technology Hefei Anhui 230009 China lxf@hfut.edu.cn

## Abstract

Rutile TiO_2_ shows great potential for photocatalytic water (H_2_O) splitting into oxygen (O_2_) and hydrogen peroxide (H_2_O_2_). However, the mechanism of surface water oxidation on rutile TiO_2_ remains unclear, involving complex ground-state thermal catalysis and excited-state photocatalysis processes. Here, by using linear response time-dependent density functional theory (LR-TDDFT), we investigate H_2_O oxidation at both the ground-state and excited-state levels. Our results show that O_2_ formation is thermocatalytic and occurs at room temperature, while H_2_O_2_ desorption is driven by photogenerated holes, requiring light to overcome a high-energy barrier, which agrees with experiments showing O_2_ formation is more favorable. Furthermore, comparing the computational results obtained using the local PBE and nonlocal HSE functionals, we find the HSE provides a more accurate description of the electronic interactions between TiO_2_ and the adsorbates, and the reaction pathways, especially under excited-state conditions. Our work provides a pathway for understanding TiO_2_ water oxidation mechanisms.

## Abstract 2

Dual mechanisms of water oxidation on rutile TiO_2_ are revealed *via* LR-TDDFT: O_2_ forms through thermocatalysis, while H_2_O_2_ desorption proceeds *via* photoexcitation to overcome a high energy barrier.

## Graphical abstract

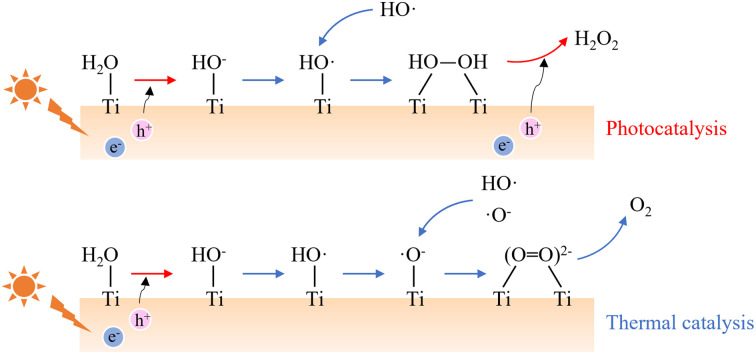

## Introduction

The water oxidation reaction is a crucial step in the photocatalytic water splitting process, enabling the production of high-value chemicals such as hydrogen peroxide (2H_2_O → H_2_O_2_ + H_2_) and oxygen (2H_2_O → O_2_ + 2H_2_).^1–4^ H_2_O_2_ exhibits widespread application in wastewater treatment, chemical synthesis, and paper manufacturing, while O_2_ is essential for photosynthesis and respiration. One of the significant challenges in water oxidation is the selective control of the reaction pathway.^5,6^ The water oxidation reaction can proceed *via* a two-electron pathway, producing H_2_O_2_ (2H_2_O + 2h^+^ → H_2_O_2_ + 2H^+^),^3,4^ or a four-electron pathway, generating O_2_ (2H_2_O + 4h^+^ → O_2_ + 4H^+^).^1,2,7^ Although the two-electron pathway requires fewer electrons, the four-electron pathway typically dominates in practical applications, resulting in reduced selectivity for H_2_O_2_.^7–9^ Titanium dioxide (TiO_2_) possesses promise in water photocatalysis due to its excellent light stability, low toxicity, and cost-effectiveness.^10–20^ However, the detailed mechanisms governing the surface water oxidation reaction remain poorly understood, particularly regarding the reaction pathways and selective control in both ground-state thermal catalytic and excited-state photocatalytic processes.

The adsorption and dissociation behavior of water on rutile TiO_2_(110) has been extensively characterized using TPD,^21^ STM,^22^ 2PPE,^23^ and IRAS^24^ techniques. These studies have established that water tends to dissociate at oxygen vacancy sites, forming bridging hydroxyls, while molecular adsorption can also occur at Ti_5*c*_ sites. Under specific conditions, dissociative adsorption at Ti_5*c*_ sites has also been observed. Moreover, UV-induced photodissociation of isolated H_2_O monomers has been experimentally confirmed at cryogenic temperatures, supporting the existence of photoresponsive surface intermediates relevant to photocatalysis.^22,25^ The oxidation of water on TiO_2_ surfaces involves multiple competing reaction pathways, with complex intermediate species. Notably, the formation of peroxide intermediates proceeds through diverse mechanisms, such as oxo–oxo coupling of OH˙or O˙^−^ radicals on metal sites,^8,26,27^ as well as nucleophilic attack by water molecules on M–O˙species.^27–29^ Subsequently, these peroxide intermediates can either undergo protonation to form H_2_O_2_ or deprotonation to yield O_2_. Normally, water oxidation prefers the formation of O_2_ in an alkaline environment, while the yield of H_2_O_2_ is poor.^7–9,30^ The intricate nature of these competing pathways presents a significant challenge in understanding the mechanisms and kinetics that govern H_2_O_2_ and O_2_ evolution.^31,32^ Furthermore, the photocatalytic water oxidation reaction involves excited-state intermediates, including OH˙radicals, O˙^−^ anions, and peroxide species.^8,33,34^ Although some strategies such as doping, surface modification, and development of novel catalysts have been proposed to improve the selectivity of H_2_O_2_ production, the yield is still far below expectations.^31,35–39^ Meanwhile, theoretical simulation of these photoinduced intermediates and their reactions on the catalyst surface remains a challenge.

Recent comparative studies have provided valuable insight into this issue. Valdés *et al.* investigated photo-oxidation on the rutile (110) surface using density functional theory (DFT), reporting a moderate overpotential of approximately 0.78 V at pH = 0, sufficiently low to permit spontaneous reaction under light irradiation.^40^ Similarly, Li *et al.* demonstrated that visible light can effectively drive the OER on anatase, with a comparable overpotential of around 0.7 V.^27^ Malik *et al.* carried out a comparative DFT study on rutile, anatase, and brookite surfaces.^5^ By constructing free energy diagrams involving key intermediates (OH*, O*, and OOH*), they showed that anatase tends to favor the two-electron pathway for H_2_O_2_ generation, whereas brookite and rutile exhibit less definitive selectivity. Specifically, brookite may promote˙OH formation *via* a one-electron pathway, while rutile shows a greater tendency toward the four-electron pathway for O_2_ evolution. Despite these advances, accurately simulating the excited-state behavior of photoinduced species (such as OH˙, O^−^, and peroxide intermediates) remains a significant computational challenge. Most previous studies have relied on ground-state DFT, which is insufficient to capture the dynamic nature of photoexcited carriers. To overcome this limitation, time-dependent DFT (TDDFT), particularly in its linear-response formulation (LR-TDDFT), has been employed to model excitation spectra and electron–hole interactions in photocatalytic systems.^41–43^ However, the commonly used PBE functional systematically underestimates band gaps and excited-state energies. Hybrid functionals such as HSE provide more accurate descriptions but are computationally demanding.^44,45^ To address this trade-off, our group has recently developed low-rank approximations^46^ that significantly accelerate hybrid LR-TDDFT calculations while retaining high accuracy.

In this work, we simulate the adsorption of two water (H_2_O) molecules on TiO_2_ surfaces leading to O_2_ and H_2_O_2_ formation, focusing on reactions with high ground-state barriers. Our results show that O_2_ formation follows a thermocatalytic pathway, while H_2_O_2_ formation is photocatalytic, requiring light to overcome a high desorption barrier. This explains why H_2_O_2_ is rarely observed in experiments. We compare the PBE and HSE functionals, finding that while PBE underestimates the excited-state reaction barrier, HSE provides a more accurate description of electronic interactions and reaction pathways. This research highlights the importance of hybrid functionals in accurately modeling excited-state photocatalytic reactions.

## Computational methods

We perform the ground-state DFT calculations implemented in the plane-wave-based Vienna *ab initio* simulation package (VASP).^47,48^ The projector-augmented wave potential is used to separate the valence electrons from the core ion.^49^ The cutoff energy is set to 500.0 eV. The exchange-correlation interaction is described by using both the PBE^50^ functional within GGA and the HSE06 (ref. 44 and 45) hybrid functional. The van der Waals interaction is included by employing the DFT-D3 method with Becke-Jonson damping.^51,52^ An 11 × 11 × 15 Monkhorst–Pack *k*-point mesh^53^ are sampled in the first Brillouin zone for geometry optimization of bulk and the *Γ* point for surface systems. We use a 4 × 2 TiO_2_ (110) supercell containing three O–Ti–O layers to simulate water (H_2_O) dimer adsorbed on the TiO_2_ (110) surface. A 20.0 Å vacuum gap is included above the surface in this model. All atoms are allowed to relax until the force acting on each is less than 0.01 eV Å^−1^. We use the VASPKIT code for post-processing of the VASP calculated data.^54^ The vibrational energies are computed using numerical Hessians *via* finite differences, as implemented in VASP by setting IBRION = 5. In this approach, the second derivatives of the total energy with respect to atomic displacements are evaluated numerically to obtain the phonon frequencies at the *Γ* point.

In order to study the transition-state electronic structures, we adopt the climbing image nudged-elastic band (CI-NEB) method^55,56^ to compute the reaction path and obtain the energy barrier in the ground-state. We perform a spin analysis for all the structures involved in the reactions and find that only structures J and K exhibit nonzero magnetic moments (Fig. 2 and Table S1), since the oxygen molecule prefers the triplet state. Therefore, we employ non-spin-polarized TDDFT calculation.

We perform the excited-state electronic structure calculations within LR-TDDFT^57,58^ implemented in the KSSOLV^59–61^ software package developed by our research group. The optimized norm-conserving Vanderbilt (ONCV) pseudopotentials, as well as PBE and HSE hybrid exchange-correlation functional are used.^62,63^ All calculations are carried out at the *Γ* point. The kinetic energy cutoff is set to 20.0 hartree. In the framework of LR-TDDFT, the excitation wavefunction (e–h pairs) is expanded in the ground-state KS orbitals *ϕ*_*i*_ as1
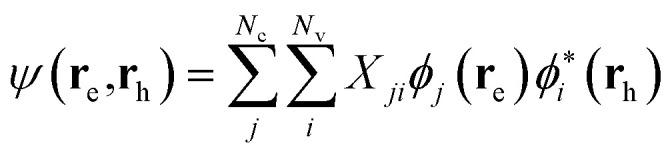
where *N*_v_ and *N*_c_ are the number of valence and conduction orbitals, *i* and *j* represent label valence and conduction orbitals, respectively. *X* are the transition vectors, and corresponding excitation energies are obtained by diagonalizing the LR-TDDFT Hamiltonian. In the LR-TDDFT, the excitation wave function is expanded over both valence and conduction orbitals, which are two-particle representation (e–h pairs) from an excitonic perspective, and can obtain both the excited-state electrons and holes at the same time.

## Results and discussion

**Fig. 1:**
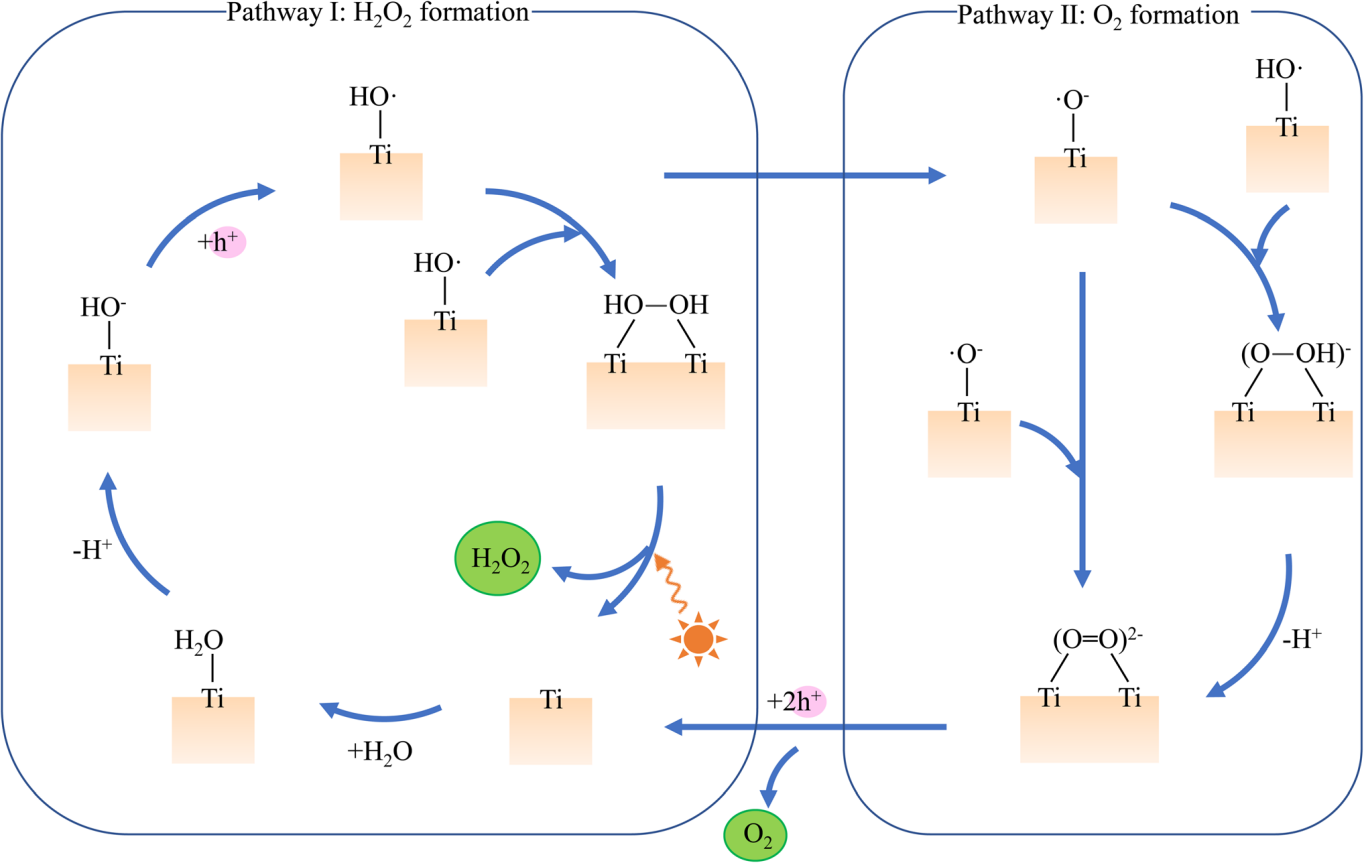
Schematic diagram of two photocatalytic pathways for two water molecules adsorbed on rutile TiO_2_ (110) surface. Pathway I shows the gradual conversion of surface hydroxyl groups to produce H_2_O_2_, and Pathway II illustrates the stepwise transformation of adsorbed oxygen species into O_2_.

**Fig. 2:**
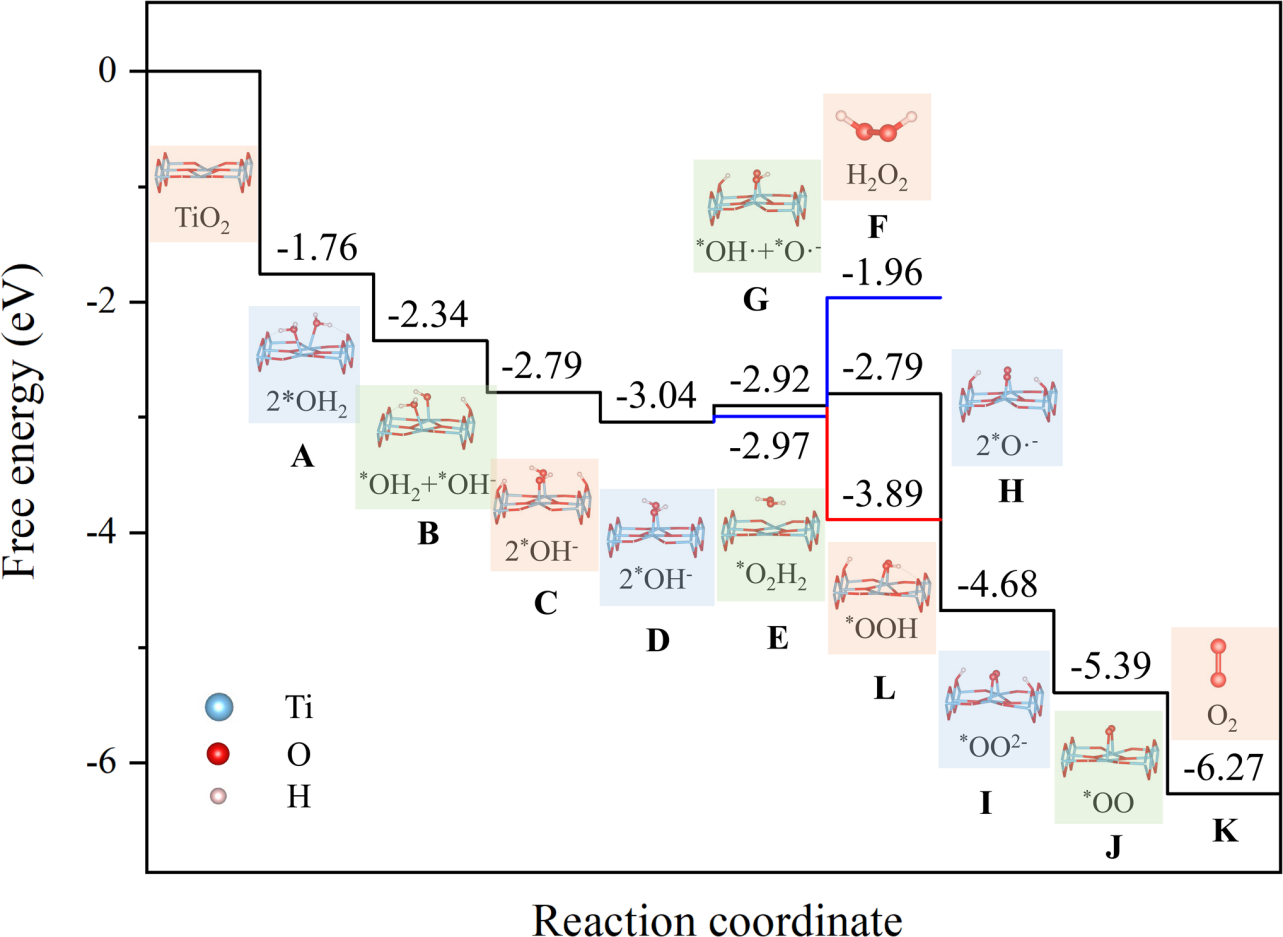
Gibbs free energy profiles for reaction path of generating O_2_ or H_2_O_2_ from two water molecules adsorption on rutile TiO_2_ (110) surface with light-induced bias potential of *U* = 2.8 V. The reaction pathway includes multiple reaction steps (a)–(l), marked along the reaction coordinate. The numbers (in eV) represent the relative free energy of each state.

To investigate the potential photocatalytic reactions of two H_2_O molecules adsorbed on the rutile TiO_2_(110) surface, we simulate two reaction pathways for the generation of hydrogen peroxide or oxygen (Fig. 1). Fig. 2 depicts their structural configurations and corresponding free energy evolution, which involves thirteen reaction steps (eqn (a) to (l)). Initially, the two H_2_O molecules are adsorbed on two adjacent titanium (Ti) atoms (Structure A). With the hydrogen (H) atoms migrating to the bridged oxygen (O_br_) site, the dissociated water adsorption structure is formed (Structure C). Two protons are released from the O_br_ site, and two holes are transferred to surface oxygen atoms near the adsorbed OH anions, resulting in the formation of two OH˙radicals (Structure D).^64,65^ The released protons are assumed to exist as solvated H^+^ species in the solution phase. The formation of H_2_ through the reduction of H^+^ by photogenerated electrons on bare TiO_2_ surfaces is generally unfavorable due to the high activation energy required for hydrogen atom migration and recombination. Multiple experimental studies indicate that H_2_ production on bare TiO_2_ surfaces is rare and typically requires metal co-catalysts (*e.g.*, Pt) or elevated temperatures.^66,67^ The desorption behavior of OH˙radicals is highly dependent on the environment. While OH˙can desorb into the gas phase under UHV conditions *via* photoinduced processes, in aqueous environments, OH˙radicals on rutile TiO_2_ surfaces predominantly remain surface-bound.^68–70^ Subsequently, the oxygen atoms from the OH groups approach each other, leading to the formation of a hydrogen peroxide adsorption structure with an energy barrier of 0.68 eV (Structure E). Hydrogen peroxide can desorb from the surface, overcoming a determinative barrier of 1.17 eV. Additionally, the two hydrogen atoms in Structure D can further migrate to the O_br_ site, bringing the oxygen atoms closer together to form an O–O bond (Structure I), with an energy barrier of 0.32 eV. In Structure G, a hydrogen atom may first be removed to form HO–O species, followed by the transfer of another hydrogen to the O_br_ site, leading to Structure I. Further, two protons are released from the O_br_ site, with two holes again transferred to the surface oxygen from the adsorbed water molecules (Structure J). The reaction concludes with the desorption of an O_2_ molecule. The relevant reaction equations and energy barriers for ground-state are as follows:

a 2H_2_O + 2* → 2*OH_2_

b 2*OH_2_ + ^Δ^ → *OH_2_ + *OH^−^ + ^Δ^H^+^, *E*_a_ = 0.02 eV

c *OH_2_ + *OH + ^Δ^H^+^ + ^Δ^ → 2*OH^−^ + 2^Δ^H^+^, *E*_a_ = 0.00 eV

d 2*OH^−^ + 2^Δ^H^+^ + 2*h*^+^ → 2^Δ^ + 2*OH˙ + 2H^+^

e 2*OH˙ → *O_2_H_2_, *E*_a_ = 0.68 eV

f *H_2_O_2_ → H_2_O_2_ + *, *E*_a_ = 1.17 eV

g 2*OH˙ → *OH˙ + *O˙^−^ + ^Δ^H^+^, *E*_a_ = 0.30 eV

h *OH˙ + *O˙^−^ + ^Δ^H^+^ → 2*O˙^−^ + 2^Δ^H^+^, *E*_a_ = 0.32 eV

i 2*O˙^−^ + 2^Δ^H^+^ → *OO^2−^ + 2^Δ^H^+^, *E*_a_ = 0.04 eV

j *OO^2−^ + 2^Δ^H^+^ + 2*h*^+^ → *OO + 2H^+^

k *OO → O_2_ + *, *E*_a_ = 0.00 eV

l *OH˙ + *O˙^−^ + ^Δ^H^+^ → *OOH^−^ + ^Δ^H^+^, *E*_a_ = 0.27 eV

i′ *OOH^−^ + ^Δ^H^+^ → *OO^2−^ + 2^Δ^H^+^, *E*_a_ = 0.01 eV

* stands for the surface Ti_5*c*_ site and Δ for the surface O_br_ site.

Subsequently, we calculated the Gibbs free energy profiles for the two reaction pathways of water oxidation on the TiO_2_ surface, leading to the formation of O_2_ and H_2_O_2_, respectively. The computational details are presented in the SI. Fig. 2 illustrates the calculated Gibbs free energy changes (Δ*G*) for the elementary steps involved in these pathways under a photo-induced external potential *U*, where *U* is defined as the energy difference between the water reduction potential and the conduction band minimum (CBM) of TiO_2_. Under visible-light irradiation (*U* = 2.8 V), all elementary steps in the O_2_ evolution pathway exhibit relatively small Δ*G* values, indicating that the formation of O_2_ is thermodynamically favorable on the TiO_2_ surface. Notably, the overall Δ*G* for the complete O_2_ evolution pathway is calculated to be −6.27 eV, which is significantly lower than the value of 4.92 eV obtained in the absence of photo-excitation.

This clearly highlights the thermodynamic advantage of photo-induced excitation in facilitating water oxidation toward O_2_. For the H_2_O_2_ formation pathway, the desorption step of H_2_O_2_ (reaction f) presents a considerably high Δ*G* of 1.01 eV, suggesting that this step is thermodynamically unfavorable. For the two water-splitting pathways yielding H_2_/O_2_and H_2_/H_2_O_2_, the total enthalpy changes are 5.92 eV and 3.98 eV, respectively. To evaluate the rationality of the proposed mechanisms, we have summed the energy changes from steps (a) through (l), obtaining total values of 5.66 eV and 3.62 eV, respectively. These are in good agreement with the theoretical values within a reasonable margin of error, confirming that the proposed reaction pathways form a complete and energetically consistent catalytic cycle.

It is important to note that the Gibbs free energy diagram presented in Fig. 2 was obtained based on an ideal, defect-free TiO_2_(110) surface. While this model provides a clean reference for exploring intrinsic reaction energetics, it does not capture the complexity of real catalytic surfaces. In particular, the presence of oxygen vacancies can significantly modify the adsorption strength and reaction pathways of water molecules.^71,72^ Previous studies have shown that water tends to dissociatively adsorb at oxygen vacancy sites, forming bridging hydroxyls that are stabilized by local structural distortions. Regarding the desorption of O_2_, our results suggest that it can occur spontaneously from the defect-free surface due to weak physisorption through van der Waals interactions.^73^ However, this behavior should not be generalized to defective surfaces. On TiO_2_ surfaces containing oxygen vacancies, O_2_ is known to chemisorb at these sites, often forming O^−^_2_ species that are considerably more strongly bound.^74^ Each oxygen vacancy can stabilize up to two O_2_ molecules, leading to strong retention of oxygen species on the surface. Consequently, spontaneous desorption of O_2_ is highly unlikely in such cases. These findings highlight that the desorption behavior of O_2_ is strongly dependent on the surface defect landscape and must be interpreted with caution when extrapolating from idealized models. Future work should systematically investigate the role of surface defects—such as oxygen vacancies, step edges, and undercoordinated sites—on the energetics and mechanisms of water oxidation reactions.

To deeply understand the photocatalytic mechanisms, we analyzed several processes with higher energy barriers under excited-states: the reaction steps in eqn (e)–(h). The higher reaction energy barriers indicate that these reactions are difficult to occur spontaneously at room temperature and may require light to activate them.

Usually, the excited carriers can quickly decay to the lowest excited-state through ultrafast non-radiative processes after photon excitation,^75,76^ so we assume that the system evolves to the lowest excited-state energy surface before e–h recombination. We compute the excited-state energies of all intermediate structures by LR-TDDFT and then add the ground-state total energy and the lowest excitation energy of each structure. It is difficult to evaluate the excited-state potential energy surface in TDDFT framework within plane-wave basis sets for condensed phase systems. Therefore, we use the ground-state reaction path approximation, ignoring the response of the nuclear system to the excited electrons.^77,78^ This approximation is reasonable for condensed matter systems, especially solid systems, and has been demonstrated to be effective in previous works.^41,42,79,80^ The main reasons are as follows. (1) Since the condensed matter system often has a large number of electrons, the excitation of a single electron has a limited influence on the system, which is often smaller than the thermal fluctuation at a certain temperature. (2) Dense energy levels create bands, making excited carriers decay quickly, leaving nuclear motion with no time to respond to these evolution of excited carriers.

**Fig. 3:**
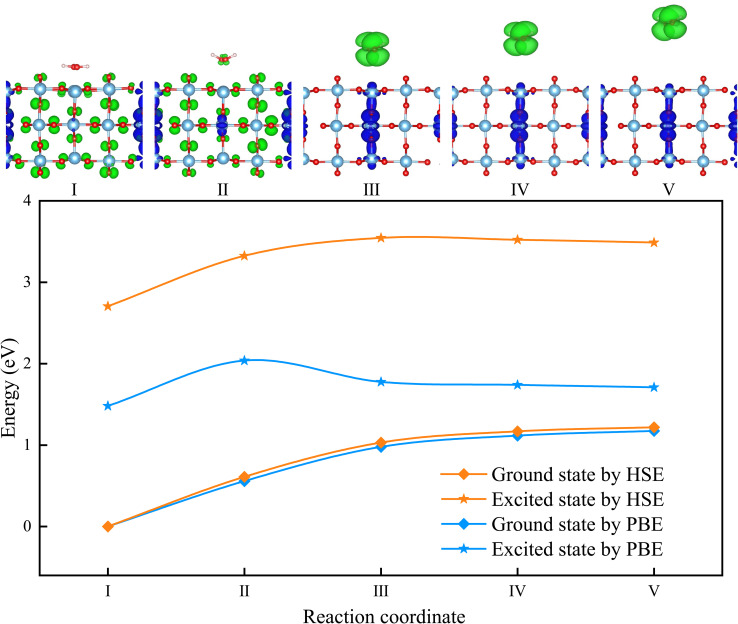
Energy profiles for the H_2_O_2_ desorption, at ground-state and the lowest excited-state levels calculated by PBE and HSE functionals. The top panels display distributions of the lowest excited-state electrons (blue) and holes (green) in real space calculated by HSE functional. The isosurface value is 0.0002 e Bohr^−3^.

Next, we examined the energy distributions of the ground- and excited-states along the reaction paths of the four previously discussed reactions calculated by PBE functional (Fig. 3 and S1–S3). We found that for the process of HO–OH bond formation (eqn (e)), the excited-state energy barrier is higher than the ground-state energy barrier, which indicates a ground-state thermal catalytic process. For the O–H bond dehydrogenation process (eqn (g) and (h)), the energy barriers for the excited-state and ground-state are similar, and the reaction can occur without the involvement of light. For the H_2_O_2_ desorption process, the excited-state energy barrier is lower than the ground-state energy barrier, suggesting that light is required for the reaction to proceed, making it an excited-state photocatalytic process. This will be the focus of our study, and we analyzed the reaction mechanism using both the PBE and HSE functionals.

As shown in Fig. 3, three intermediate structures are introduced between the reactant (designated as Structure I) and the product (designated as Structure V). For ground-state, the energy differences between PBE and HSE calculations are small, with reaction barriers of 1.17 eV and 1.22 eV, respectively, which manifests this reaction cannot proceed at room temperature without photon assistance. Both functionals show that the energy gradually increases along the reaction path, without any significant transition state features. In contrast, for the excited-state, the energy differences at PBE and HSE functionals are substantial, with excited-state energies calculated by HSE functional significantly higher than those calculated by PBE functional. The reaction barriers in the excited-state level are 0.56 eV for PBE and 0.79 eV for HSE, both significantly lower than their respective ground-state barriers. Although the HSE-calculated excited-state energy barrier is higher than that obtained from PBE, we believe it is more physically reasonable. The PBE functional tends to underestimate excitation energies and reaction barriers due to its inherent self-interaction error and tendency to over-delocalize electrons, especially in systems involving charge-transfer-like excited states.^81,82^ In contrast, the HSE hybrid functional incorporates a portion of exact exchange, which corrects for delocalization errors and yields a more localized and realistic description of the excited-state electron density. This reduction in the excited-state barrier highlights the critical role of photogenerated holes in facilitating the H_2_O_2_ desorption process.

Moreover, the excited-state energy profiles feature the reaction transition states, where the transition states are located at Structure II for PBE functional and Structure III for HSE functional. Despite these differences in energy, the exciton distributions calculated using the two functionals are remarkably similar. By analyzing the exciton distributions, we find that the photogenerated holes and electrons in the lowest excited-states between Structure I and Structure II are localized within the TiO_2_ lattice, indicating that these excited-states are intrinsic excitations of TiO_2_. Conversely, in Structures III and V, the photogenerated holes are predominantly localized in H_2_O_2_. This declares that redistribution of holes to H_2_O_2_ as the reaction progresses activates the reaction and lowers the energy barrier. To assess the likelihood of photoexcitation relevant to the desorption process, we analyzed the excited-state density of states (DOS), as shown in Fig. S4. A significant density of excited states is observed in the range of 2.70–3.00 eV, with a pronounced peak near 2.90 eV. This indicates the presence of a large number of accessible unoccupied states within the energy window typically covered by visible light sources. The presence of these states suggests that photoinduced electronic transitions are probable under experimentally relevant excitation conditions.

**Fig. 4:**
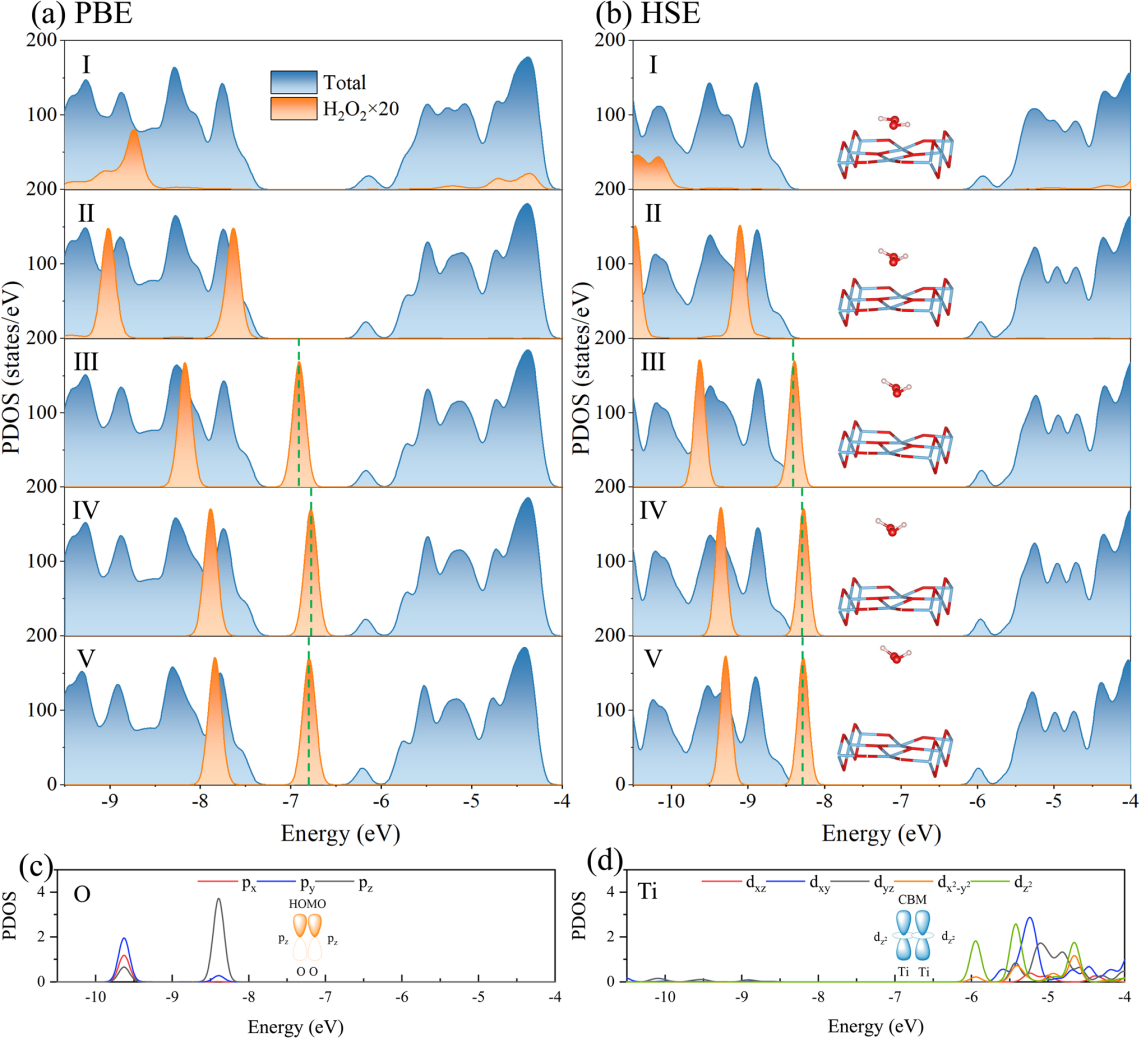
Total and projected density of states (TDOS and PDOS) for the reactant, intermediates, and product structures in the H_2_O_2_ desorption process, calculated using (a) PBE and (b) HSE functionals. (c and d) PDOS of O atoms of H_2_O_2_ and Ti atoms of TiO_2_, calculated by the HSE functional for Structure III. Inserts illustrate the spatial distribution and overlap of the orbitals that contribute to the interaction between H_2_O_2_ and the TiO_2_ surface. The vacuum level is set to zero in each panel.

In order to understand the evolution of photoexcited carriers in the desorption of H_2_O_2_, we calculated the projected density of states (PDOS) changes at the PBE and HSE06 levels, as shown in Fig. 4. In Structures I and II, the states of H_2_O_2_ are primarily located at lower energy regions below the Fermi level, without significant interaction with the valence band maximum (VBM) of TiO_2_. This indicates that the excitations at this stage are intrinsic to TiO_2_ and are unrelated to the adsorbate. In case of Structure III, HSE calculations show a noticeable upward shift of the H_2_O_2_ states approaching the VBM, compared with PBE calculation. It can be seen that the highest occupied molecular orbital (HOMO) of H_2_O_2_ exhibits significant energetic overlap with the VBM of TiO_2_, which indicates an enhanced ability of H_2_O_2_ to capture photogenerated holes, thus enabling the reaction to achieve the transition state. As shown in Fig. 4(d), the VBM is primarily composed of the p_*z*_ orbital of the O atom of H_2_O_2_, while the CBM consists of the d^2^_*z*_ orbital of the lattice Ti atoms. Furthermore, the bonding characteristics of the HOMO of H_2_O_2_ and the VBM of TiO_2_ are presented in Fig. 4(c). In contrast, PBE calculations reveal the emergence of new occupied states within the TiO_2_ band gap, which are contributed by H_2_O_2_, create a novel excitation pattern and lead to a significant reduction in the calculated excitation energy. Consequently, Structure II is identified as the excited-state transition state in the PBE calculations (Fig. 3). In Structure IV, the H_2_O_2_ states continue to shift upward, becoming the dominant active states. This indicates a transition from intrinsic excitations of TiO_2_ to H_2_O_2_-dominated electronic excitations. Meanwhile, the differences between PBE and HSE become more pronounced, with HSE providing a more accurate depiction of the upward shift and the dominant role of H_2_O_2_ states. In Structure V, the H_2_O_2_ states move even closer to the Fermi level, indicating further reduction in excitation energy, ultimately facilitating the desorption reaction. Overall, the PDOS changes depicted in Fig. 4 reveal the dynamic evolution of the electronic structure of TiO_2_ and H_2_O_2_ during the desorption process. The PBE calculations introduce new occupied states within the TiO_2_ band gap, resulting in significantly lower calculated excitation energy barriers compared to HSE. This discrepancy not only reflects the differences in the capabilities of the two functionals in describing excited-state electronic structures but also provides critical theoretical insight into the role of photogenerated holes in activating the reaction and reducing the energy barrier.

## Conclusions

In summary, we comprehensively simulate pathways from two H_2_O molecules to H_2_O_2_ or O_2_ from the ground and excited-state perspective, revealing that O_2_ formation is thermocatalytic and occurs readily at room temperature, while H_2_O_2_ formation is photocatalytic due to a high ground-state energy barrier, requiring light excitation. This explains the rarity of experimental H_2_O_2_ detection. Local PBE and nonlocal HSE functionals significantly impact excited-state calculations, with PBE underestimating the band gap and reaction barriers by 0.23 eV, whereas HSE provides more accurate electronic interactions and excited-state pathways. While ground-state electronic structures show minor differences, excited-state barriers exhibit notable discrepancies. This work highlights the importance of precise excited-state modeling for photocatalyst design, offering insights into TiO_2_-based systems and guiding efforts to improve solar-driven energy conversion, such as green hydrogen production.

## Author contributions

W. Hu conceived the idea for this study and designed the research. L. Wang conducted the research. X. Liu helped with the analysis and interpretation of the data. L. Wang wrote the manuscript and all authors assisted with editing, analysis, and interpretation.

## Conflicts of interest

The authors declare no competing financial interest.

## Acknowledgements

## Supplementary Material

SC-016-D5SC02736E-s001

SC-016-D5SC02736E-s002

SC-016-D5SC02736E-s003

SC-016-D5SC02736E-s004

SC-016-D5SC02736E-s005

SC-016-D5SC02736E-s006

## Data Availability

The data supporting this article have been included as part of the SI. Calculation details of Gibbs free energy, magnetic moments of the structures involved in the reaction path, energy profiles for ground-state and the lowest excited-state of step eqn (e), (g) and (h) calculated by using PBE functional, as well as the lowest excited-state electrons and holes in real space of the reactant, intermediates, and product structures during these processes. See DOI: https://doi.org/10.1039/d5sc02736e.
