# Supplementary material for: Revealing the photocatalytic dissociation of water molecules on rutile TiO2 surface via hybrid functional based linear response time-dependent density functional theory
Source: Chem Sci. 2025 Aug 22;16(36):16876–84. doi: 10.1039/d5sc02736e (PMC12371566; doi:10.1039/d5sc02736e)
Supplement: SC-016-D5SC02736E-s002 [file SC-016-D5SC02736E-s002.pdf]

Supplementary Information (SI) for Chemical Science.
This journal is © The Royal Society of Chemistry 2025

Supporting Information

Revealing the photocatalytic dissociation of water molecules on rutile TiO_2 surface via hybrid functional based linear response time-dependent density functional theory

Lei Wang,[a] Xiaofeng Liu,*[b] Qunxiang Li,*[a] Jinlong Yang,[a] and Wei Hu*[a]

[a] *Department of Chemical Physics, and State Key Laboratory of Precision and Intelligent Chemistry, University of Science and Technology of China, Hefei, Anhui 230026, China*

[b] *School of Physics, Hefei University of Technology, Hefei, Anhui 230009, China*

1 Gibbs free energy calculation

The Gibbs free energy for each reaction step can be calculated according to the following equations:

$$\Delta G_a = E(2^*\mathrm{OH_2}) - 2E(\mathrm{H_2O}) - E(2^*) + \Delta ZPE_a - T\Delta S_a$$
$$\Delta G_b = E(^*\mathrm{OH_2}) + E(^*\mathrm{OH^-}) + E(^\Delta\mathrm{H^+}) - E(2^*\mathrm{OH_2}) + \Delta ZPE_b - T\Delta S_b$$
$$\Delta G_c = E(2^*\mathrm{OH^-}) + E(2^\Delta\mathrm{H}^) - E(^*\mathrm{OH_2}) - E(^*\mathrm{OH}) - E(^\Delta\mathrm{H^+}) - E(^\Delta) + \Delta ZPE_c - T\Delta S_c$$
$$\Delta G_d = E(2^\Delta) + E(2^*\mathrm{OH}\cdot) + E(H_2) - E(2^*\mathrm{OH^-}) - E(2^\Delta\mathrm{H^+}) + \Delta ZPE_d - T\Delta S_d - 2U_h$$
$$\Delta G_e = E(^*\mathrm{O_2H_2}) - E(2^*\mathrm{OH}\cdot) + \Delta ZPE_e - T\Delta S_e$$
$$\Delta G_f = E(\mathrm{H_2O_2}) + E(^*) - E(^*\mathrm{H_2O_2}) + \Delta ZPE_f - T\Delta S_f$$
$$\Delta G_g = E(^*\mathrm{OH}\cdot) + E(^*\mathrm{O}\cdot^-) + E(^\Delta\mathrm{H^+}) - E(2^*\mathrm{OH}\cdot) + \Delta ZPE_g - T\Delta S_g$$
$$\Delta G_h = E(2^*\mathrm{O}\cdot^-) + E(2^\Delta\mathrm{H^+}) - E(^*\mathrm{OH}\cdot + ^*\mathrm{O}\cdot^- + ^\Delta\mathrm{H^+}) + \Delta ZPE_h - T\Delta S_h$$
$$\Delta G_i = E(^*\mathrm{OO^{2-}}) + E(2^\Delta\mathrm{H^+}) - E(2^*\mathrm{O}\cdot^-) + E(2^\Delta\mathrm{H^+}) + \Delta ZPE_i - T\Delta S_i$$
$$\Delta G_j = E(^*\mathrm{OO}) + E(H_2) - E(^*\mathrm{OO^{2-}}) + E(2^\Delta\mathrm{H^+}) + \Delta ZPE_j - T\Delta S_j - 2U_h$$
$$\Delta G_k = E(\mathrm{O_2}) + E(^*) - E(^*\mathrm{OO}) + \Delta ZPE_k - T\Delta S_k$$
$$\Delta G_l = E(^*\mathrm{OOH^-}) + E(^\Delta\mathrm{H^+}) - E(^*\mathrm{OH}\cdot) + E(^*\mathrm{O}\cdot^-) + E(^\Delta\mathrm{H^+}) + \Delta ZPE_l - T\Delta S_l$$
$$\Delta G_{i'} = E(^*\mathrm{OO^{2-}}) + E(2^\Delta\mathrm{H^+}) - E(^*\mathrm{OOH^-}) + E(^\Delta\mathrm{H^+}) + \Delta ZPE_{i'} - T\Delta S_{i'}$$

where E represents the single-point energy, and U_h denotes the external potential for photogenerated holes. ZPE is the zero-point energy and S is the vibrational entropy. The ZPE corrections and entropy contributions were obtained from vibrational frequencies. The entropy contributions for adsorbed species are defined by considering only their vibrational motion, as described below:

$$S = k_B \ln \prod_{i=1}^{N} \frac{1}{1 - \exp\left(\frac{\hbar\omega_{X,i}}{k_B T}\right)} \tag{1}$$

where k_B is the Boltzmann constant, $\hbar$ is the Planck constant, ω is the wavenumber, i represents a specific vibrational mode, and N is the total number of vibrational modes for the adsorbed specie X. The entropic contributions for gaseous molecules are taken from standard thermodynamics tables, with the temperature taken as 300 K.

Table 1 Magnetic moments of the structures involved in the reaction path.

Structure ID	Magnetic Moment (μ_B)
O, A, B, C, D, E, F, G, H, I, L	0.00
J	2.00
K	2.00

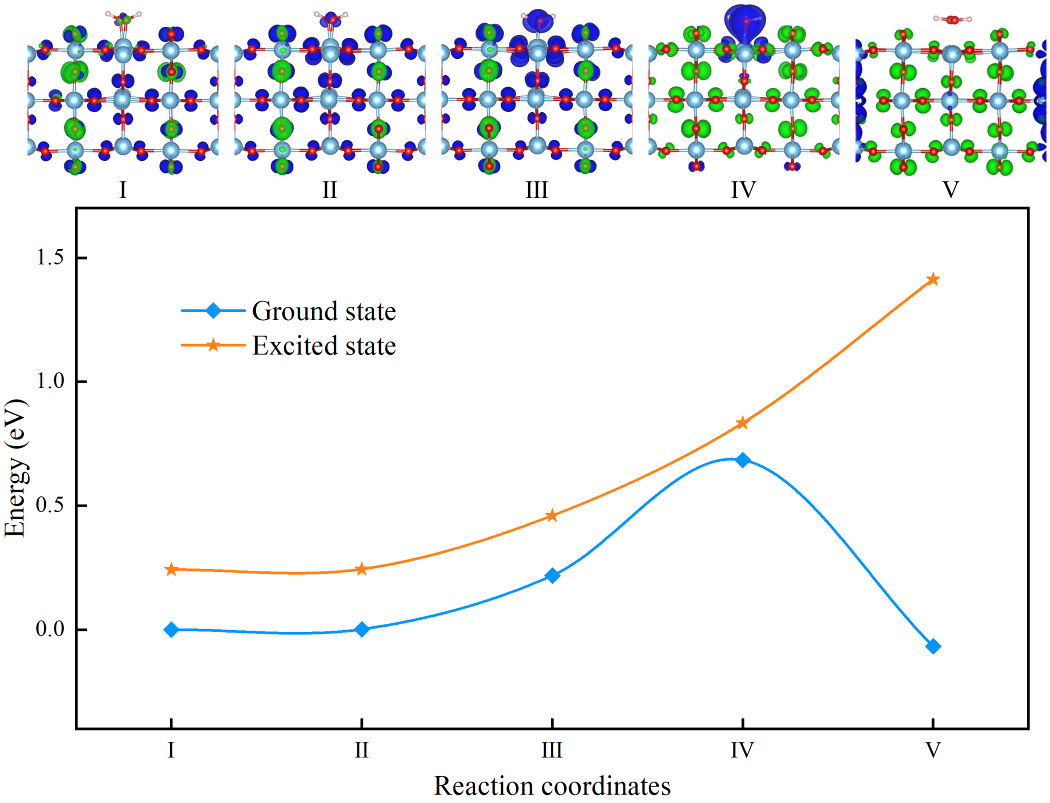

Figure 1 Energy profiles for the ground-state and the lowest excited-state in the reaction step of Equation e calculated by using PBE functional. The top panels display distributions of the lowest excited-state electrons (blue) and holes (green) in real space. The isosurface value is 0.0002 e·Bohr^{-3}.

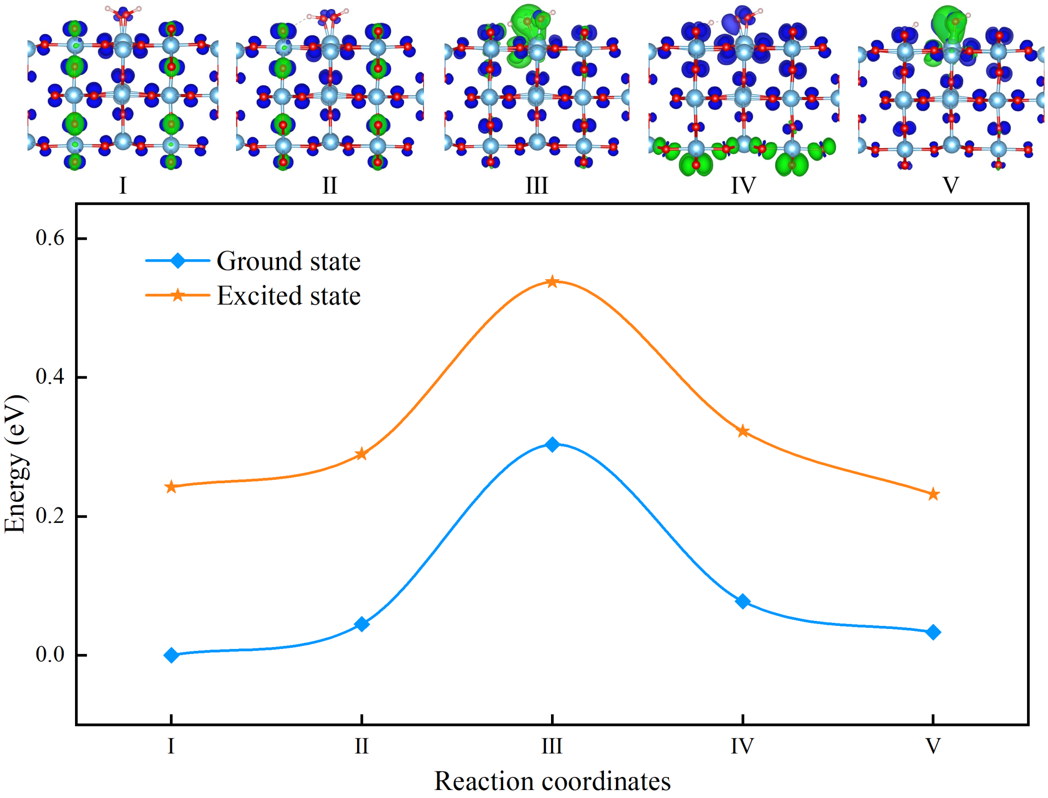

Figure 2 Energy profiles for the ground-state and the lowest excited-state in the reaction step of Equation g calculated by using PBE functional. The top panels display distributions of the lowest excited-state electrons (blue) and holes (green) in real space. The isosurface value is 0.0002 e·$Bohr^{-3}$.

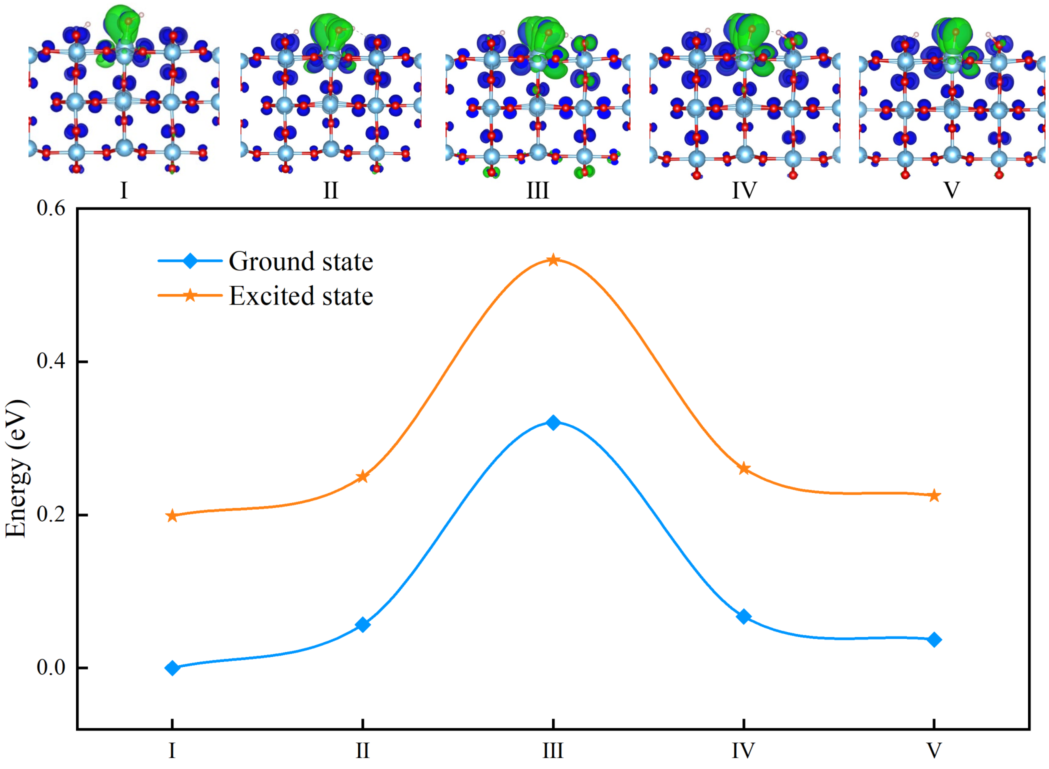

Figure 3 Energy profiles for the ground-state and the lowest excited-state in the reaction step of Equation h calculated by using PBE functional. The top panels display distributions of the lowest excited-state electrons (blue) and holes (green) in real space. The isosurface value is 0.0002 e·Bohr^{-3}.

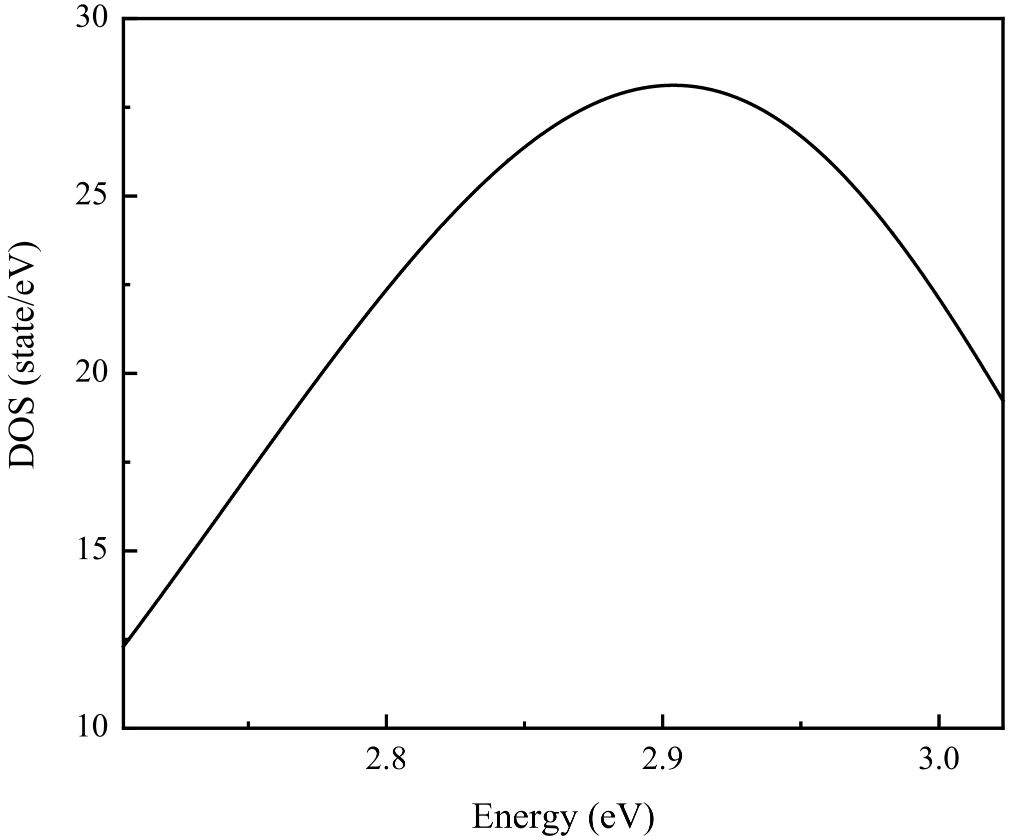

Figure 4 Excited-states DOS for H_2O_2 adsorption on TiO_2 (Structure G) by HSE06 functional.
