# Supplementary figures and images for: Revealing the photocatalytic dissociation of water molecules on rutile TiO2 surface via hybrid functional based linear response time-dependent density functional theory
Source: Chem Sci. 2025 Aug 22;16(36):16876–84. doi: 10.1039/d5sc02736e (PMC12371566; doi:10.1039/d5sc02736e)

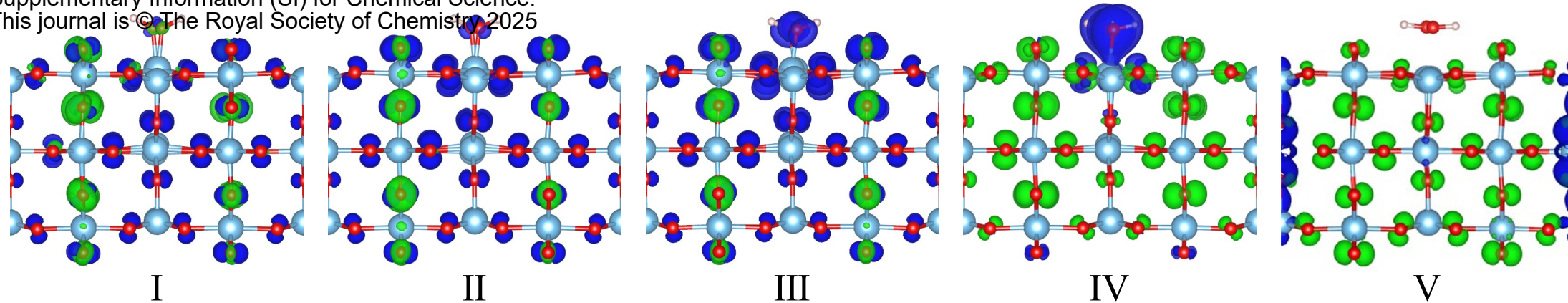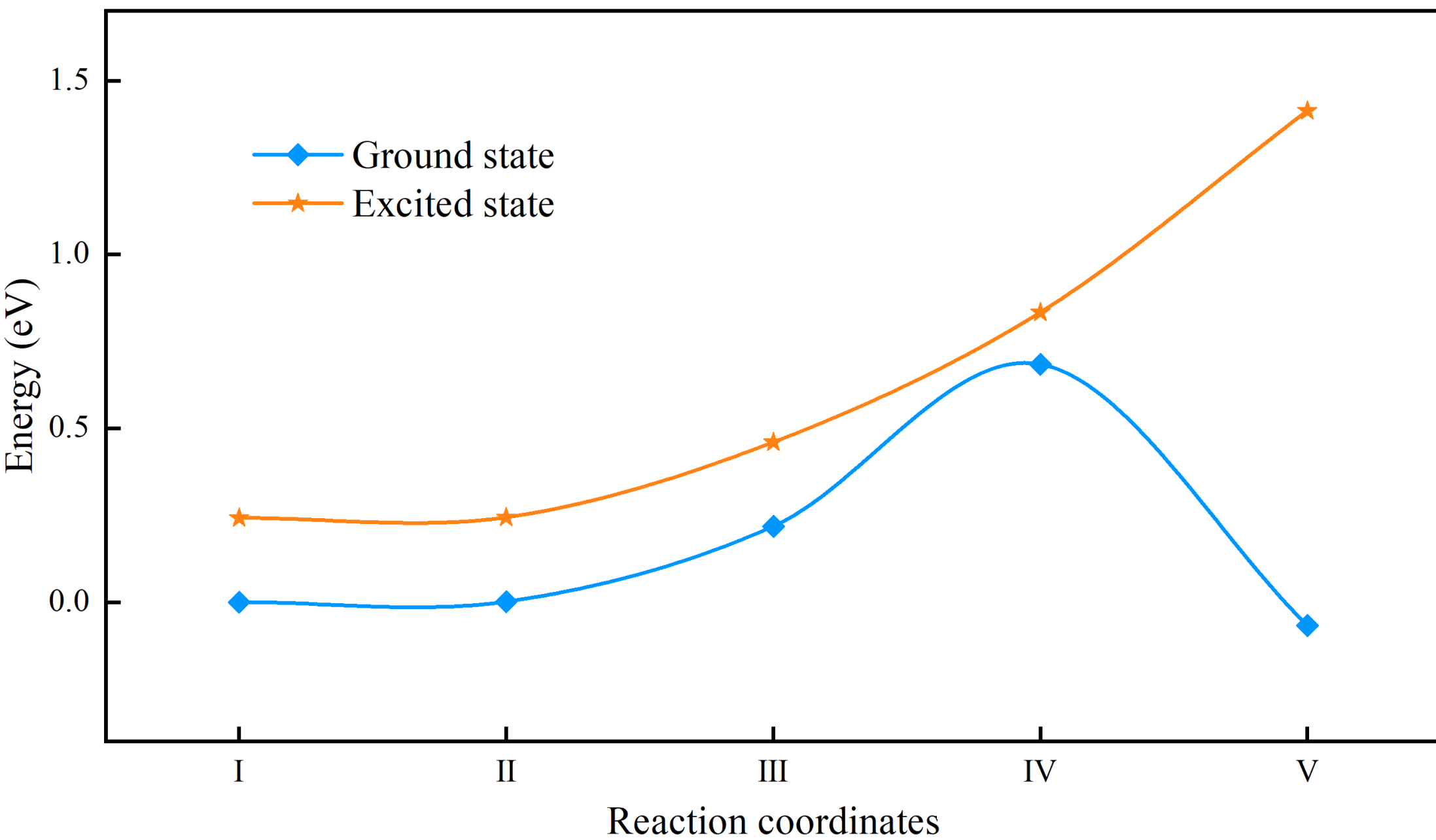

Supplement: SC-016-D5SC02736E-s003 [file SC-016-D5SC02736E-s003.pdf]

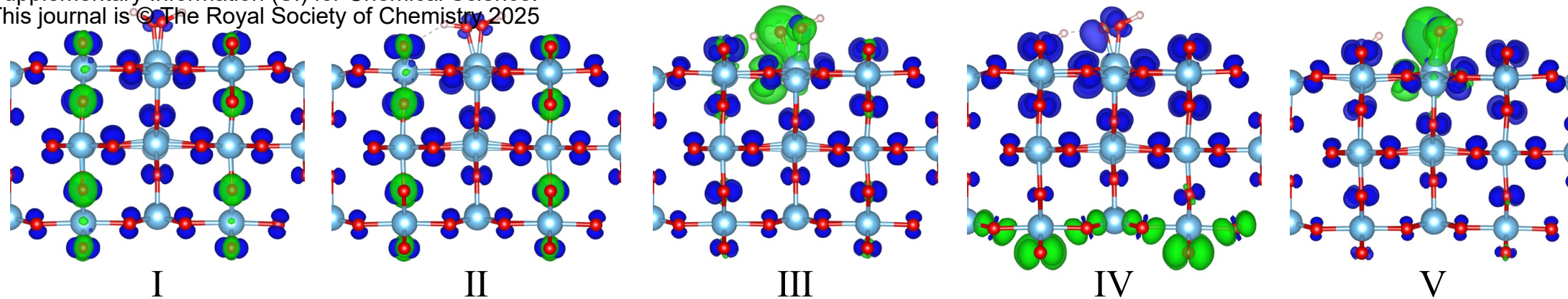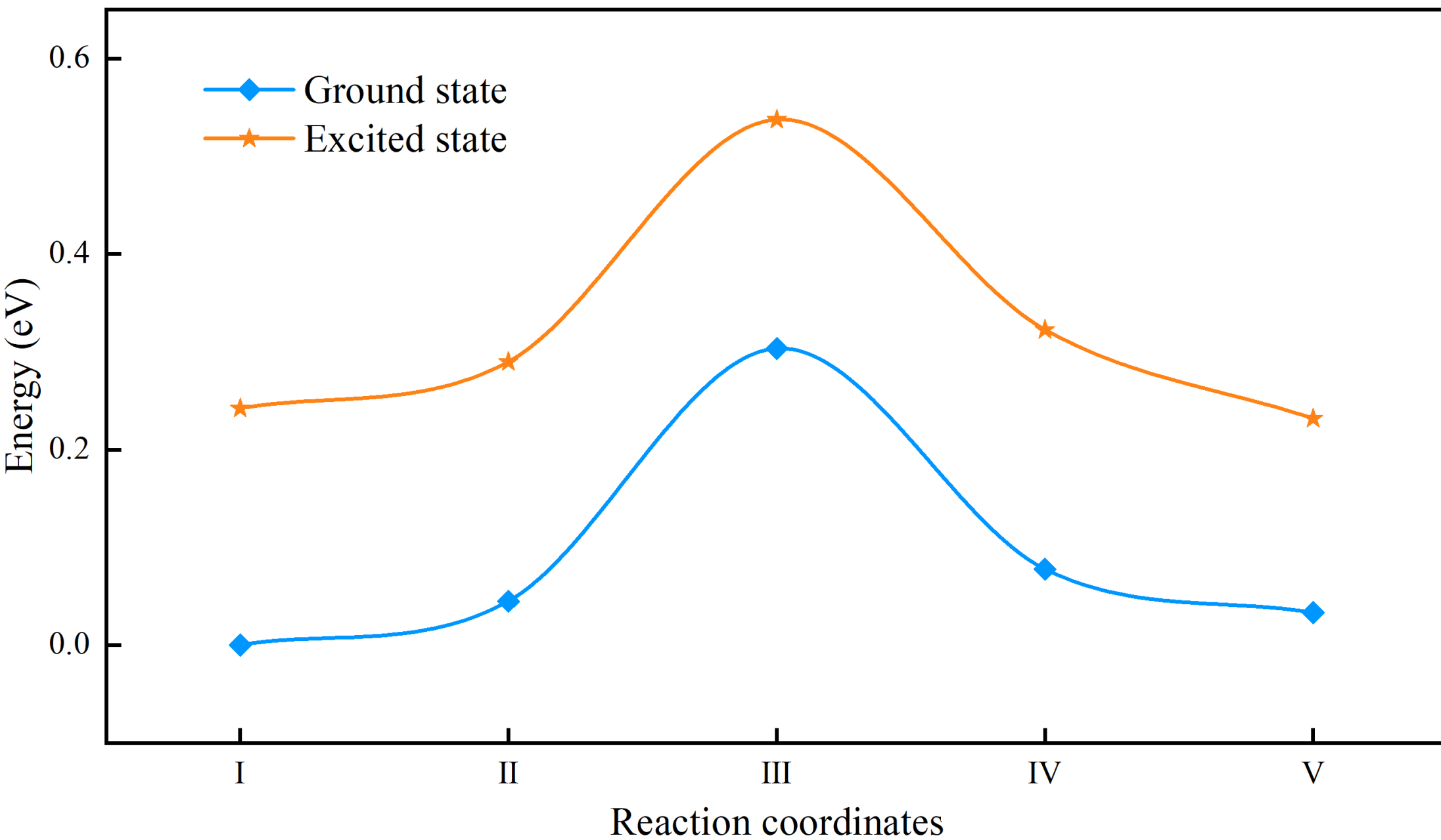

Supplement: SC-016-D5SC02736E-s004 [file SC-016-D5SC02736E-s004.pdf]

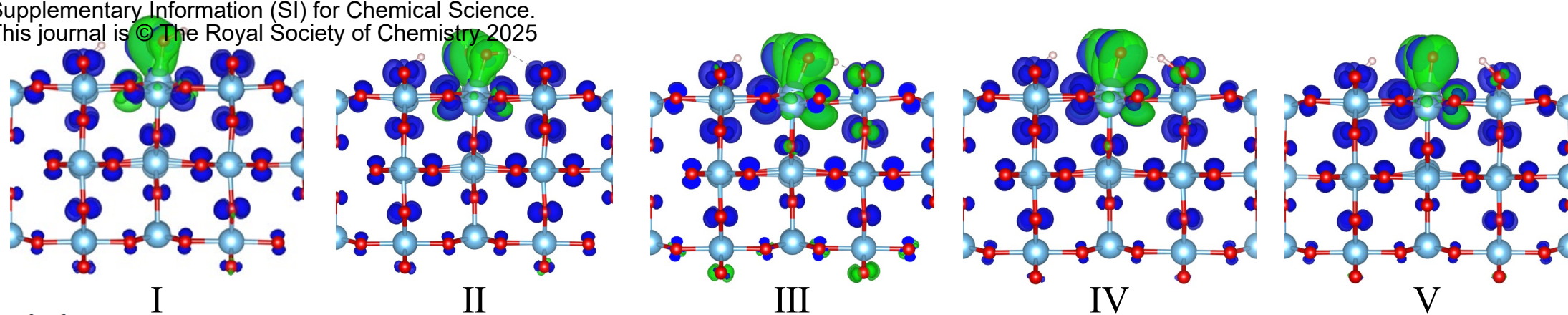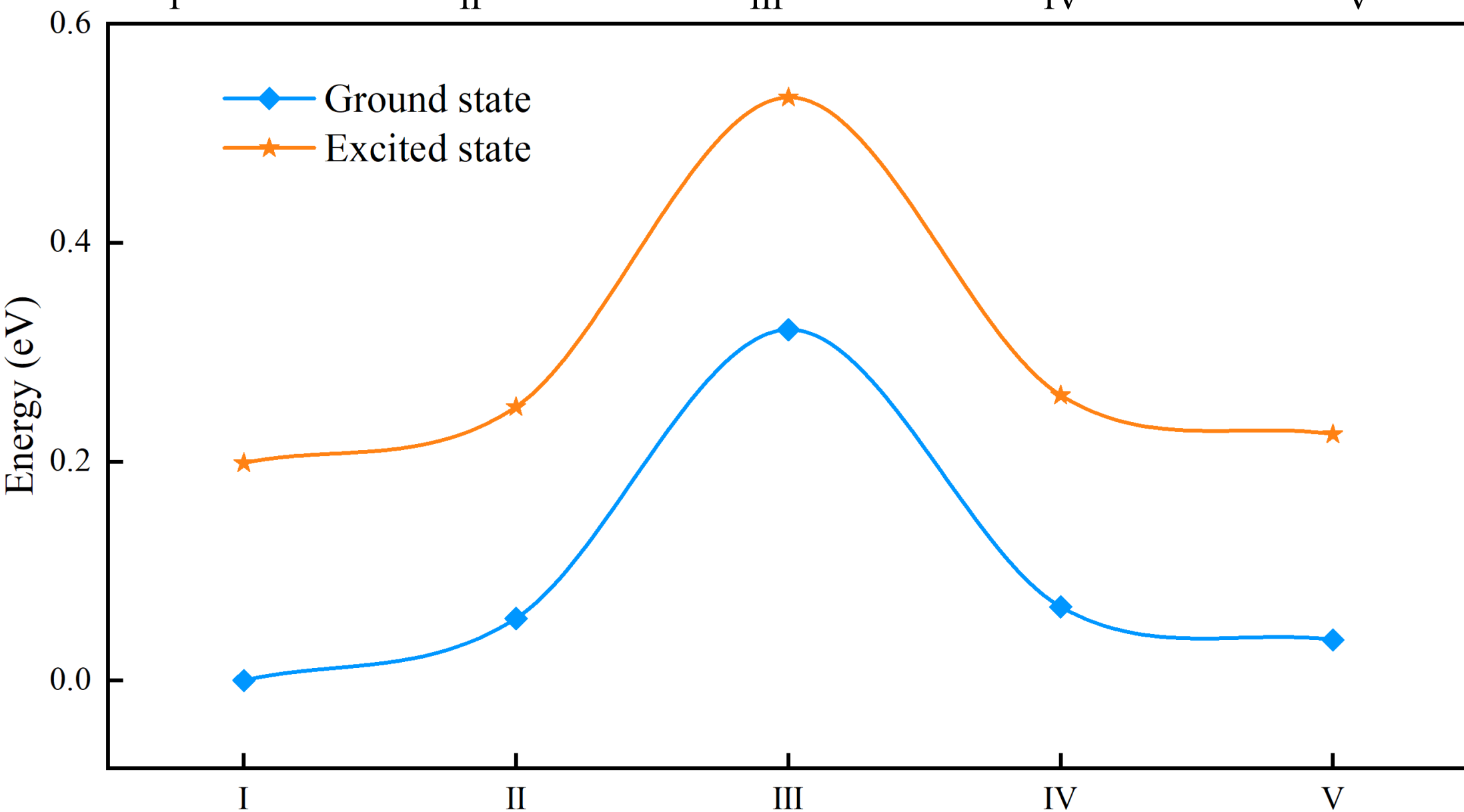

Supplement: SC-016-D5SC02736E-s005 [file SC-016-D5SC02736E-s005.pdf]

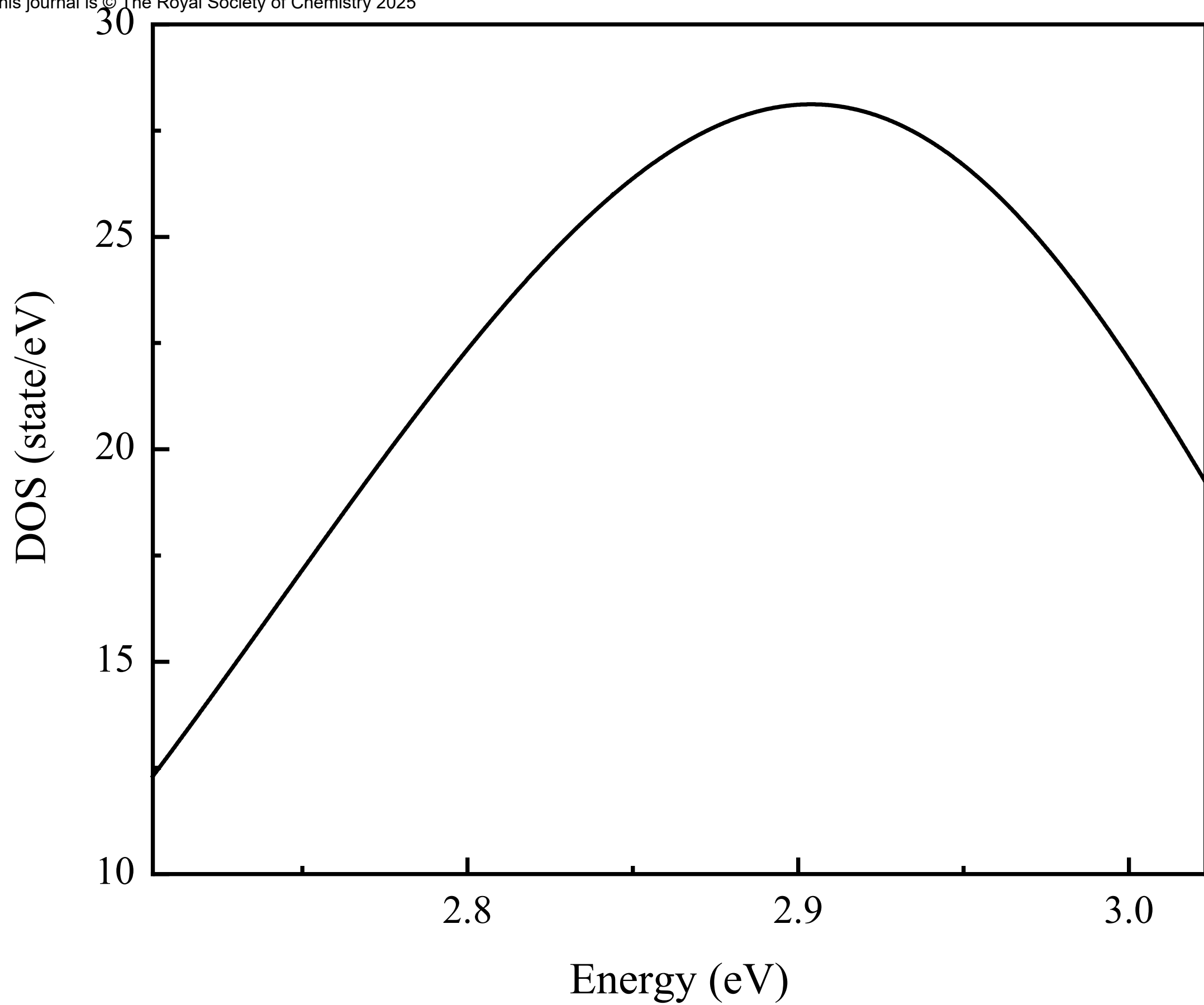

Supplement: SC-016-D5SC02736E-s006 [file SC-016-D5SC02736E-s006.pdf]
